# Nanoparticles and Chemical Inducers: A Sustainable Shield against Onion White Rot

**DOI:** 10.3390/biology13040219

**Published:** 2024-03-27

**Authors:** Ahmed Mohammed Elenany, Mahmoud Mohammed Mohammed Atia, Entsar E. A. Abbas, Mahmoud Moustafa, Mohammed O. Alshaharni, Sally Negm, Ahmed Saeed Mohammed Ali Elnahal

**Affiliations:** 1 Plant Pathology Department, Faculty of Agriculture, Zagazig University, Zagazig 44511, Egypt; ahmedelenany@zu.edu.eg (A.M.E.);; 2 Department of Biology, College of Science, King Khalid University, Abha 61413, Saudi Arabia; 3 Department of Life Sciences, College of Science and Art Mahyel Aseer, King Khalid University, Abha 62529, Saudi Arabia

**Keywords:** onion white rot, *Sclerotium cepivorum*, nanoparticles, inducers, fungicides, eco-friendly management

## Abstract

**Simple Summary:**

This study explores alternative methods to combat onion white rot, a destructive disease caused by *Sclerotium cepivorum*, which poses a significant threat to onion cultivation, leading to substantial yield losses. Traditional fungicides, while effective, raise environmental concerns, prompting a search for eco-friendly alternatives. This research evaluates the efficacy of nanoparticles and chemical inducers in managing the disease. Nanoparticles, including Fe_3_O_4_, Cu, and ZnO, exhibit promising results in inhibiting the growth of the pathogen both in vitro and in vivo. Additionally, chemical inducers like salicylic acid show potential in reducing disease incidence and severity while promoting plant growth. This study also includes an assessment of traditional fungicides’ inhibitory effects on *S. cepivorum*. Overall, this research provides valuable insights into eco-friendly strategies for onion white rot management, offering potential solutions to mitigate the impact of this devastating disease on onion crops and the environment.

**Abstract:**

This study investigated the effectiveness of nanoparticles and chemical inducers in managing onion white rot caused by *Sclerotium cepivorum*. The pathogen severely threatens onion cultivation, resulting in significant yield losses and economic setbacks. Traditional fungicides, though effective, raise environmental concerns, prompting a shift toward eco-friendly alternatives. In this study, four *S. cepivorum* isolates were utilized, each exhibiting varying degrees of pathogenicity, with the third isolate from Abu-Hamad demonstrating the highest potency. During the in vitro studies, three nanoparticles (NPs) were investigated, including Fe_3_O_4_ NPs, Cu NPs, and ZnO NPs, which demonstrated the potential to inhibit mycelial growth, with salicylic acid and Fe_3_O_4_ NPs exhibiting synergistic effects. In vivo, these nanoparticles reduced the disease incidence and severity, with Fe_3_O_4_ NPs at 1000–1400 ppm resulting in 65.0–80.0% incidence and 80.0–90.0% severity. ZnO NPs had the most positive impact on the chlorophyll content, while Cu NPs had minimal effects. At 1000 ppm, Fe_3_O_4_ NPs had variable effects on the phenolic compounds (total: 6.28, free: 4.81, related: 2.59), while ZnO NPs caused minor fluctuations (total: 3.60, free: 1.82, related: 1.73). For the chemical inducers, salicylic acid reduced the disease (10.0% incidence, 25.0% to 10.0% severity) and promoted growth, and it elevated the chlorophyll values and enhanced the phenolic compounds in infected onions. Potassium phosphate dibasic (PDP) had mixed effects, and ascorbic acid showed limited efficacy toward disease reduction. However, PDP at 1400 ppm and ascorbic acid at 1000 ppm elevated the chlorophyll values and enhanced the phenolic compounds. Furthermore, this study extended to traditional fungicides, highlighting their inhibitory effects on *S. cepivorum*. This research provides a comprehensive comparative analysis of these approaches, emphasizing their potential in eco-friendly onion white rot management.

## 1. Introduction

Onions (*Allium cepa* L.), ubiquitous in kitchens worldwide, stand as a culinary cornerstone, adding depth and flavor to many dishes. Studies have highlighted their richness in phytochemicals, particularly medicinal flavonoids [1]. Onions hold substantial economic importance globally and are paramount in Egypt for both local consumption and export. However, recent years have witnessed a significant decline in onion production, primarily attributed to the devastating white rot disease caused by *S. cepivorum* [2]. This continual threat poses a persistent risk to *Allium* spp. cultivation, triggering disease outbreaks wherever such crops are grown.

The disease displays noticeable symptoms above ground, including wilting, yellowing of older leaves, and die-back of leaf tips. Eventually, collapse and decay occur as the symptoms advance along the leaf blades. Concurrently, below the ground, root infection leads to watery decay and the appearance of fluffy, white mycelial growth at the bulb base. Numerous sclerotia form within these mycelial mats, serving as the main source of inoculum for the next cultivated crops [3]. This pathogen severely threatens onion cultivation, leading to significant yield losses and economic setbacks. It has become widely prevalent in Egypt, leading to severe damage and, at times, complete crop loss of up to 100%, as noted by Ahmed and Ahmed [4]. Notably, this pathogenic menace is a major constraint on onion cultivation, particularly in Upper Egypt [5]. Researchers exploring strategies to manage white rot disease consider both efficacy and environmental implications, with traditional fungicides serving as a reliable but not always eco-friendly solution [6]. Among the alternatives are nanoparticles and chemical inducers, such as salicylic acid, which have demonstrated promising results in managing plant diseases [7,8].

Nanoparticles, with their minute size and unique properties, offer a novel avenue for disease management. Recent studies have highlighted the potential of nanoparticles in inhibiting the growth and spread of pathogens, including *S. cepivorum* [3]. When strategically employed, these microscopic wonders can target the pathogen while minimizing the impact on non-target organisms and the environment. In addition, nanoparticles can stimulate morphological and physiological changes in plants, with their efficacy influenced by factors like the composition, size, surface properties, reactivity, and dosage [9]. The concentration of NPs is crucial for effectiveness, and nanotechnology presents unique applications in biotechnology and agriculture [10]. NPs can serve as targeted carriers, delivering substances like herbicides, pesticides, fertilizers, or genes to specific cellular organelles in plants [11]. Despite the potential benefits, inadequate information exists on NP toxicity in plants, and few studies have explored the mechanisms underlying NPs’ influences on plant growth. Understanding NP action’s physiological, biochemical, and molecular mechanisms is essential for optimizing their impact on host growth [12]. The World Health Organization (WHO) and the United States Environmental Protection Agency (USEPA) set limits for heavy metals in drinking water: 5 mg/L for Zn, 0.3 mg/L for Fe, and 2 mg/L for Cu by the WHO; 1.3 mg/L for Cu by the USEPA [13].

Inducers, particularly salicylic acid, have become key players in plant defense mechanisms. Salicylic acid is a signaling molecule that activates the plant’s immune responses, fortifying its defenses against invading pathogens [14]. Incorporating these chemical inducers into disease management strategies holds promise, providing a targeted and potentially sustainable approach to combating white rot in onions. The shift toward nanoparticle and chemical inducer-based approaches represents a conscious effort to balance disease control and ecological responsibility. By exploring these alternatives, we aim to safeguard onion crops and cultivate a more sustainable and environmentally conscious agricultural landscape.

This study aims to compare various strategies for managing onion white rot disease caused by *S. cepivorum*. These strategies include the application of nanoparticles (Fe_3_O_4_ NPs, Cu NPs, and ZnO NPs), chemical inducers (salicylic acid, PDP, ascorbic acid), and traditional fungicides. Specifically, our objectives encompass evaluating the effectiveness of these alternative methods in inhibiting the mycelial growth of *S. cepivorum* in vitro, assessing their ability to reduce the disease incidence and severity in infected onions under greenhouse conditions, and investigating their impact on key plant health parameters, including the chlorophyll content (a, b, and carotenoids) and phenolic compounds (total, free and bound), in infected onions under various management strategies. Furthermore, we aim to compare the efficacy of nanoparticle-based treatments, chemical inducers, and traditional fungicides to identify the most promising eco-friendly management strategies for onion white rot. Through this comprehensive analysis, we seek to provide insights into sustainable agriculture by offering recommendations for adopting effective and environmentally friendly management practices for onion white rot disease.

## 2. Materials and Methods

### 2.1. Pathogenicity Test

Four isolates of *S. cepivorum* were obtained from infected onion plants in the districts/regions of Zagazig 30°35′09.1″ N, 31°31′16.3″ E, Minia Elkamh 30°30′49.8″ N, 31°20′28.8″ E, Abu-Hamad 30°32′00.8″ N, 31°40′17.0″ E, and Abu-Kabeer 30°43′19.3″ N, 31°40′17.3″ E. These isolates were evaluated for pathogenicity using 50-day-old onion seedlings (cultivar “Giza 20, red”). By autoclaving in 500 mL glass bottles with 100 g of barley grains and 50 mL of water, fungus inoculum was created. After being infected with five discs of each strain, the barley medium was cultured for 30 days at 20 ± 2 °C. The fungus inoculum was added to 30 cm plastic pots containing sanitized sand–clay soil at a ratio of 1:1 (*v*/*v*) for seven days prior to transplanting, with a rate of 3–5 g/kg. Standard agricultural practices were followed.

At 100 days post transplantation (dpt), the onions were removed to assess the illness’s frequency and severity. The following formula was used to compute the disease incidence (%) according to Brix and Zinkernagel [15]:

$$Plant\;infection\;rate\;\%=\frac{number\;of\;infected\;plants}{number\;of\;plants\;overall}\times 100$$

On a scale from 0 to 4, the severity of the disease was ranked as follows: 0 represented healthy plants; 1 was slightly severe (leaf yellowing and reduced root system); 2 was moderately severe (bulbs, wing and die-back of leaves, badly decayed root system); 3 represented severe disease (full plant yellowing, leaf die-back, semi-watery soft rot of scales and roots); and 4 represented greatly severe disease (fully dead plants, extensively decayed bulbs and roots). With slight modifications, the method proposed by Zewide et al. [16] was used to determine the severity of the disease:

$$Disease\;severity=\frac{\sum (Disease\;scale\times Number\;of\;plants\;in\;each\;scale)}{Total\;number\;of\;plants\times maximal\;disease\;scale}\times 100$$

**Figure 1:**
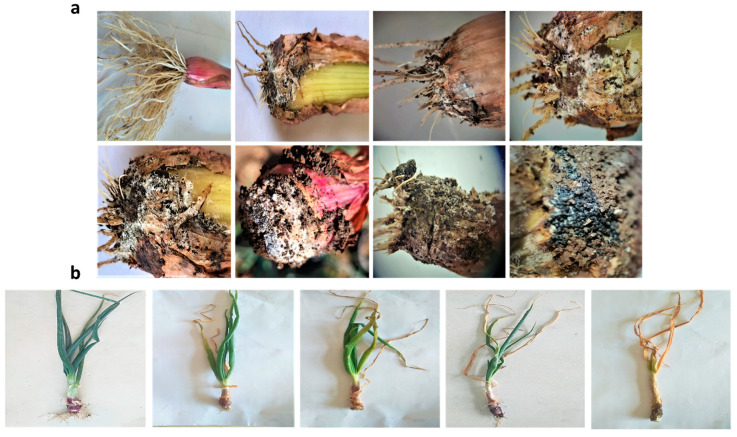
(**a**) The modified scale was applied to pulps, and the examination under a stereomicroscope revealed infected, rotten bulbs covered with Whitty mycelium and small black–brown sclerotia. (**b**) A modified disease severity scale was employed to assess the impact on onion plants.

This scale was modified as shown in Figure 1. Isolate 2 was chosen because it was the most aggressive and had the greatest pathogenicity of all the tested isolates. It was used to determine the severity of the white rot disease in the onion seedlings.

### 2.2. Nanoparticle Preparation and Characterization

This study synthesized various nanoparticles (NPs), including iron as magnetite, copper metal, and zinc oxide, each with distinct concentrations and well-defined nanometric sizes. Notably, magnetite was coupled with salicylic acid (SA) at a concentration of 100 ppm. The entire process of designing, preparing, and characterizing these nanometric compounds was meticulously carried out at the Nanotechnology and Advanced Materials Central Lab, Agriculture Research Center, Giza, Egypt.

#### 2.2.1. Copper Metal Nanoparticle (Cu NP) Preparation 

The resulting copper NPs underwent characterization through powder X-ray diffraction (XRD) and transmission electron microscopy (TEM), using a 200 kV TecnaiG2 20 X-Twin model from FEI Company, Amsterdam, The Netherlands. Ascorbic acid played a dual role, acting as an antioxidant for colloidal copper by scavenging free radicals and reactive oxygen molecules. Polyvinylpyrrolidone functioned as both a size controller and a polymeric capping agent, preventing nuclei aggregation through its polar groups and forming coordination bonds with the copper NPs on the surface [17].

Copper NPs possess significant appeal for various applications, particularly regarding biocompatibility [18]. Chemical reduction was chosen as the synthesis method due to its convenience, speed, cleanliness, and cost-effectiveness. The experimental process was executed in an inert medium with low precursor concentrations.

Initially, 1 g of Polyvinylpyrrolidone (PVP) was dissolved in distilled deionized water, serving as an inert medium. Subsequently, 20 mL of 1% PVP was added to the solution, along with approximately 0.25 g of CuSO_4_·H_2_O, and the mixture was stirred for 60 min, resulting in a blue color. A separate solution was prepared by adding 0.2 g of ascorbic acid to 20 mL of 1% PVP, exhibiting a greenish–yellow color upon mixing with the copper sulfate solution. Furthermore, 0.8 g of NaOH dissolved in 20 mL of 1% PVP was introduced, and after stirring for 60 min, reaching a pH of 12, the color transitioned to opaque orange and then dark. The final step involved the addition of a sodium borohydride solution (0.076 g in 20 mL of 1% PVP), resulting in a black color after vigorous stirring for 20 min. The Cu NPs were subsequently separated, washed with deionized water through centrifugation, and dried either in a vacuum oven at 80 °C for 3–4 h or under nitrogen gas. The resulting black yield of Cu NPs was characterized for subsequent application [17].

#### 2.2.2. Preparation of Zinc Oxide Nanoparticles (Zn NPs)

Zinc nitrate and sodium hydroxide were precursors in a wet chemical process to create zinc oxide nanoparticles (Zn NPs), with sodium borohydrate serving as a stabilizing agent. The procedure, adapted from Behera [19] with some modifications, began by dissolving approximately 2.79 g of zinc nitrate hexahydrate [Zn (NO_3_)_2_·6H_2_O] in 100 mL of distilled deionized water through vigorous stirring for one hour. A solution of 0.48 g of sodium hydroxide (NaOH) in 60 mL of distilled deionized water was then prepared, and 1 mL of 1% NaBH_4_ was added after the dissolution of zinc nitrate. Sodium hydroxide solution was slowly added dropwise to the mixture using a burette, stirring overnight for 12 h to achieve a reduced nanosize. The resulting precipitate was left to settle, then dried in an oven at 80 °C after being filtered under suction and repeatedly cleaned with distilled dH_2_O. The obtained zinc hydroxide (Zn (OH)_2_) was transformed into ZnO through calcination in a muffle at 500 °C, yielding the desired smaller-sized ZnO nanoparticles.

#### 2.2.3. Magnetite Nanoparticle (Fe NP) Preparation 

In synthesizing biocompatible magnetite nanoparticles (Fe NPs), the hydrothermal method was employed, as outlined by Xuan [20]. This approach produced water-soluble Fe_3_O_4_ nanocrystals, capped with C_6_H_6_O_6_ (the oxidation state of ascorbic acid), facilitating their easy dispersion in hydrated aqueous systems. The resulting product underwent thorough characterization through X-ray powder diffraction (XRD) (Shimadzu XRD-6100, Kyoto, Japan), particle size analyzer, and transmission electron microscope (TEM). For the procedure, about 0.25 g of ferric chloride hexahydrate (FeCl_3_·6H_2_O) was dissolved in 25 mL of distilled deionized water, followed by the addition of 0.60 g of sodium bicarbonate (NaHCO_3_) dissolved in 10 mL of distilled deionized water. The color turned turbid brown, and upon introducing 0.12 g of ascorbic acid to the mixture, the color changed to clear black. After stirring for 15 min, the solution was completed to 50 mL with distilled deionized water and subjected to hydrothermal treatment at 160 °C for 3 h in a stainless-steel autoclave. The resulting solution was then filtered, separated, and dried at 50 °C to yield the magnetite nanoparticles (Fe NPs).

#### 2.2.4. Magnetite Nanoparticles Conjugated with Salicylic Acid (SA + Fe NPs) Preparation

In this step, a specified amount of Fe NPs was combined with 100 ppm SA and subjected to sonication, exploiting the electrostatic force between the organic compound SA and the inorganic nanomaterial Fe NPs. To maintain the activity of the Fe NPs and prevent oxidation, 0.02 g of ascorbic acid was incorporated into the sonicated solution. Notably, the crystalline nature of SA, with a melting point of 157–159 °C and a pH of 2.4, precludes direct conjugation with Fe NPs during preparation. The reagents involved in this process included Fe_3_O_4_ (prepared as per the previous illustration), salicylic acid, and ascorbic acid. The procedure encompassed carefully weighing 0.08 g of Fe NPs, dispersing them in a known volume of distilled deionized water, and subsequently adding 0.20 g of ascorbic acid and 0.30 g of salicylic acid. This mixture underwent sonication until it achieved a well-dispersed state and was ready for further applications.

#### 2.2.5. Laboratory Measurements for Nano-Scale Identification

In the laboratory, the characterization of the ZnO, Cu, and magnetite (Fe_3_O_4_) nanoparticles (NPs), along with their nanometric diameters, was conducted through various techniques. In solution form, the samples were placed as drops on a copper grid and introduced into the TEM. Liquid nitrogen was utilized to maintain the pathway beams, and the electron beam was launched to penetrate the samples for measurement.

For determining the chemical composition and purity degree, XRD analysis was performed using the X-Pert Pro model from PANalytical Company, Almelo, The Netherlands. Powdered samples were placed on a holder, filling the cavity to a depth of approximately 2 mm. As the device initiated, X-rays penetrated the samples, and the dispersion was received by a detector moving to record each angle. The data obtained were analyzed using a library system to identify specific components.

The particle size and zeta potential analysis were conducted using the Malvern Zeta Sizer Nano, Worcestershire, UK. Samples in solution form were placed in a cuvette, and each sample was adjusted to achieve transparency, minimizing the errors in the reading. The cuvette was then inserted into the device, which initiated the measurement of the particle diameters and determination of the homogeneity extent through the polydispersed index (PDI). The PDI ranges from 0 to 1, with values approaching zero indicating increased homogeneity.

### 2.3. Antifungal Activity of NPs In Vitro and In Vivo

In the in vitro antifungal activity assay, various concentrations (25, 50, and 100 μg/L ppm) of metallic NPs were introduced into PDA media and a control group devoid of treatment [21,22]. A single disc (0.5 cm diameter) of PDA fungal culture (14 days old) was centrally positioned in each petri dish, and the incubation was carried out at 20 ± 2 °C, with three replicates for each condition. Fungal growth inhibition was quantified utilizing the following formula: inhibition % = (dc − dt)/dc × 100, where dc represents the average diameter of linear growth in the control, and dt signifies the average diameter of linear growth in the treatment.

In the in vivo study, onion transplants/seedlings underwent immersion in various concentrations (1000, 1200, and 1400 ppm) of nanoparticle solutions for 5 min [21,22]. Additionally, the stem bases of the onions were subjected to two rounds of spraying with each NP at 6-week intervals from the sowing date. Parameters related to disease, disease assessment, and plant growth were recorded as previously mentioned.

### 2.4. Antifungal Activity of Chemical Inducers In Vitro and In Vivo

In the in vivo phase of the study, various chemical inducers, including salicylic acid (SA), potassium dibasic phosphate (PDP), and ascorbic acid, were applied alongside untreated and farm treatment groups. These chemicals, sourced from Al-Gomhoria Company (Cairo, Egypt), were sprayed at a concentration of 1 g/2 L of water for all the treatments. As detailed by Agha [23], inducers such as potassium dibasic phosphate (PDP) and ascorbic acid were tested at concentrations ranging from 500 to 1400 ppm, while the SA concentrations varied from 250 to 1000 ppm [24,25]. Onion transplants were immersed in each inducer, followed by two stem-base sprayings of each inducer at 6-week intervals from sowing. The disease, its assessment, and plant growth parameters were evaluated, following the previously mentioned formulae.

### 2.5. Evaluation of Different Fungicides In Vitro and In Vivo

In the assessment of the effect of different fungicide concentrations on the in vitro linear growth, three systemic fungicides (Table S1), namely Tolclofos-methyl, Bellis^®^ 38% WG Pyraclostrobin + Boscalid, Topsin M, Thiophanate methyl 70% WP, and BOGARD Teubconazole250 g/L, were tested at concentrations of 1, 10, and 50 ppm [26]. The fungicides were added to PDA medium in volumes of 1 mL to achieve the desired concentrations, and both treated and untreated (control) media were distributed into three Petri dishes for each treatment. Following solidification, the Petri dishes were inoculated with 5 mm discs of *S. cepivorum*’s 14-day-old culture and then incubated at 20 ± 1 °C. The daily measurement of the linear growth of *S. cepivorum* continued until the control plates were completely filled. The reduction in *S. cepivorum* growth was calculated using the following formula: growth reduction (%) = [(C − T)/C] × 100, where C represents the average growth area (cm^2^) of *S. cepivorum* in the control, and T is the average growth area (cm^2^) of *S. cepivorum* in the treatment, as detailed by Darwesh et al. [27].

The in vivo application treatments involved dipping onion transplants into each fungicide for 5 min. Additionally, the stem bases of the onions were sprayed twice with the recommended dose of each fungicide at 6-week intervals from the sowing date. Subsequent to the fungicide application, the disease parameters, disease assessment, and plant growth parameters were evaluated following the procedures mentioned earlier.

### 2.6. Photosynthetic Pigment Evaluation

At the harvesting time, which was conducted 100 days after the transplanting date, the bulbs were harvested and their weight was measured in kilograms per plot to determine the yield, expressed as kg/pot. In the physiological analysis, pure acetone extracted photosynthetic pigments, namely chlorophyll a, chlorophyll b, and carotenoids, from fresh leaves (0.1 g). The filtrate obtained after filtration was utilized for spectrophotometric analysis of the photosynthetic pigments using a Beckman 640D spectrophotometer (Beckman Coulter, CA, USA). The measurements were conducted at wavelengths of 662 nm, 644 nm, and 440.5 nm for chlorophyll a, chlorophyll b, and carotenoids, respectively. The calculation of the pigments was carried out using Wettestein’s formula [28,29], with the following equations:

Chlorophyll a (chl. a) = (9.784 × *E_662_*) − (0.99 × *E_644_*) (µg/mL) (1)

Chlorophyll b (chl. b) = (21.426 × *E_644_*) − (4.65 × *E_662_*) (µg/mL) (2)

Carotenoids = (4.695 × *E_440_*) − 0.268 × (chl. a + chl. b) (µg/mL) (3)

Here, *E* represents the optical density reading at the specified wavelengths, and the concentrations of the pigments were expressed in mg/gm fresh weight of leaves.

### 2.7. Total, Free and Bound Phenolic Compound Evaluation

The quantification of the phenolic compounds involved the assessment of the free, bound, and total phenols utilizing the colorimetric method outlined by Snell and Snell [30]. The preparation of the Folin Denis reagent, as per Gutfinger [31], involved the addition of 100 g sodium tungstate and 25 g sodium molybdate to 700 mL distilled water. Subsequently, 50 mL phosphoric acid (85%) and 100 mL HCL (32%) were added to the mixture, shaken well in a reflex condenser and gently boiled for 10 h in a water bath. After cooling, 25 g lithium sulfate, 50 mL distilled water, and 3 to 4 drops of bromine were incorporated. The final mixture was completed to one liter. For extraction, 5 g of plant samples was stored in 25 mL of 95% ethanol in brown bottles, left in the dark for one month at room temperature, and subsequently subjected to an air current until dry. The dried extracts were then transferred into 10 mL of 10% isopropanol and stored in vials at 1 °C.

To determine the concentration of total phenols, 0.25 mL of HCL (32%) was introduced to 1 mL of the sample extract, followed by boiling in a water bath for 10 min. After cooling, 1 mL of Folin Denis reagent and 6 mL of Na_2_CO_3_ (20%) were added. The color density was measured at 520 nm using a special spectropolarimeter, with tannic acid serving as the standard compound within a range of 0–400 ppm.

The concentration of bound phenols was calculated as the difference between the total and free phenol concentrations. To establish a standard curve, 1 g of catechol was dissolved in distilled water, and various volumes of catechol solution were taken, raised to 100 mL with distilled water, and treated as previously employed in the determination of free phenols. The relationship between the reading at 520 nm and the known catechol concentrations was plotted to create the standard curve.

### 2.8. Statistical Analysis

Statistical analysis was conducted using the software Statistix 8.1 to assess the significance of the various treatments through analysis of variance (ANOVA). The Least Significant Difference (LSD) test was employed to compare the treatments and ascertain any significant differences, with the significance level set at *p* < 0.05.

## 3. Results

### 3.1. The Pathogenicity of the Causative Agent of Onion White Rot Disease

Four isolates of *S. cepivorum*, obtained from different locations (Zagazig, Minia Elkamh, Abu-Hamad, and Abu-Kabeer), were evaluated for their pathogenicity on the Giza 20 onion cultivar under controlled greenhouse conditions (Table 1). The disease incidence and severity were assessed as a percentage, while the plant growth parameters, including the fresh and dry weight, were measured in grams. *S. cepivorum* isolate three from Abu-Hamad exhibited the highest disease incidence (80.0%) and severity (100%), indicating its strong pathogenicity. This isolate also significantly reduced both the fresh weight (6.32 g) and dry weight (3.48 g) of the onion plants. Isolate one from Zagazig and isolate two from Minia Elkamh showed similar disease incidence and severity (60.0% and 87.0%, 83.0%, respectively), with slightly differing effects on the plant growth parameters. Isolate four from Abu-Kabeer demonstrated a disease incidence of 65.0% and severity of 85.0%, affecting the plant weights at intermediate levels (Table 1). The control group, with no infection, had the highest fresh and dry weights at 70.77 g and 13.10 g, respectively (Table 1).

### 3.2. Characterization of Nanoparticles

This study synthesized and characterized metallic nanoparticles (NPs) using advanced techniques, including Cu NPs, Zn NPs, Fe NPs, and SA + Fe NPs. Characterization of the Cu NPs (Figure S1) revealed well-dispersed, nearly spherical particles with an average size of approximately 50 ± 5 nm, confirmed by TEM and DLS. Zeta potential analysis showed a stable value of −23.9 mV, and XRD analysis indicated the cubic structure (card number 04-013-9963) of the as-prepared Cu NPs.

The characterization of the as-prepared ZnO NPs (Figure S2) revealed well-dispersed, nearly spherical particles with a size of approximately 80 ± 5 nm, as observed in the TEM image. Additional confirmation via dynamic light scattering (DLS) showed an average particle size of 80 ± 5 nm with a polydispersed index (PDI) of 0.552, indicating a narrow size range. Zeta potential analysis indicated a surface charge of −11.5 mV, suggesting moderate stability. XRD analysis displayed peaks corresponding to the hexagonal crystal structure of ZnO NPs (card number 01-078-2585).

Characterization of the Fe_3_O_4_ NPs (Figure S3) revealed well-dispersed, nearly spherical particles with a size of about 50 ± 5 nm, as depicted in the TEM image. Dynamic light scattering (DLS) confirmed an average particle size of 50 ± 5 nm, with a polydispersed index (PDI) of 0.431, indicating a narrow size range. Zeta potential analysis showed a surface charge of −21.0 mV, indicating moderate stability. XRD analysis displayed peaks corresponding to the rhombohedral crystal structure of Fe_3_O_4_ NPs (card number 04-0120-7038).

The characterization of the SA + Fe NPs in Figure S4 involved multiple attempts, but the most successful method was the mechanical stirring of magnetite and SA with the antioxidant agent ascorbic acid. This process relied on the electrostatic force between magnetite and SA. TEM analysis illustrated the aggregation between them, showcasing the alterations in the magnetite NPs. For further validation of the conjugation, zeta potential measurements were conducted to assess the surface charge changes. Individually, SA exhibited a zeta potential of −11.9 mV, magnetite displayed −21.0 mV, and when both SA and magnetite were combined, the zeta potential was measured at 5.78 mV.

### 3.3. The Impact of Various Nanoparticles

#### 3.3.1. In Vitro Assessment of NPs on Linear Growth Reduction

Three types of nanoparticles—Fe_3_O_4_ NPs, Cu NPs, and ZnO NPs—were tested at three various concentrations (25, 50, 100 μg/L) (Table S2, Figure S5A). Additionally, a combination of salicylic acid with Fe_3_O_4_ NPs was included. The results show that increasing the concentration of Fe_3_O_4_ NPs led to a progressive reduction in linear growth, with the highest concentration (100 μg/L) causing an 18.89% reduction. Cu NPs and ZnO NPs also exhibited dose-dependent inhibition, with the 100 μg/L concentrations resulting in substantial reductions of 47.89% and 23.33%, respectively. Salicylic acid in combination with Fe_3_O_4_ NPs demonstrated notable efficacy, with a 57.78% reduction at 100 μg/L. The control group had the highest linear growth and zero reduction percentage. The impact of nanoparticles on mycelial malformation was observed under a light microscope (Figure S5B) and stereomicroscope (Figure S5C).

#### 3.3.2. Impact of Nanoparticles on Disease Assessment and Plant Growth Parameters

**Figure 2:**
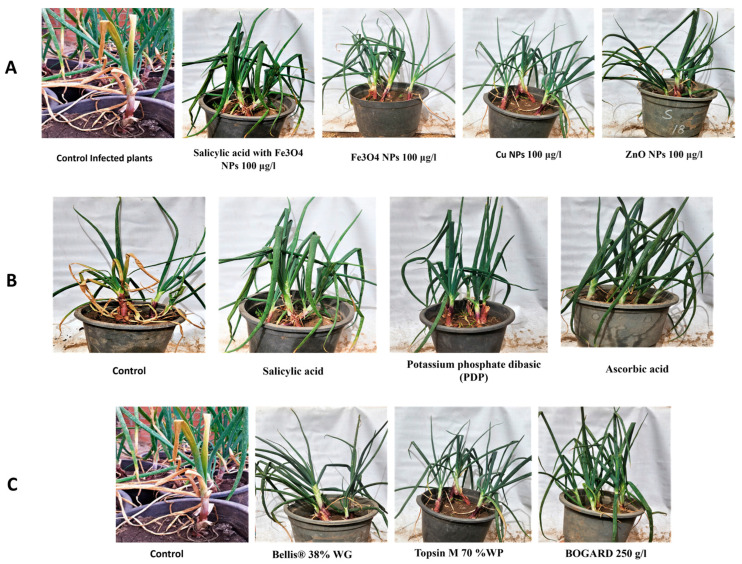
In vivo assessment. (**A**) The impact of nanoparticles was assessed. (**B**) The influence of chemical inducers, including salicylic acid, PDP, and ascorbic acid. (**C**) The in vivo impact of three tested fungicides, including Bellis, Topsin M, and BOGARD.

Various nanoparticles’ effects on the disease and plant growth in *S. cepivorum*-infected onions are shown in Table 2 and Figure 2A. Fe_3_O_4_ NPs (1000, 1200, 1400 ppm) resulted in a disease incidence of 65.0% to 80.0% and severity of 80.0% to 90.0%. The onion fresh weight ranged from 36.60 g to 37.85 g, and the dry weight from 3.85 g to 4.85 g. Cu NPs (1000 ppm, 1200 ppm, 1400 ppm) had a disease incidence of 5.0% to 25.0% and severity of 10.0% to 15.0%. The fresh weight ranged from 49.31 g to 50.98 g, and the dry weight from 7.05 g to 7.28 g. ZnO NPs (1000 ppm, 1200 ppm, 1400 ppm) showed a disease incidence of 25.0% to 30.0% and severity of 25.0% to 40.0%. The fresh weight ranged from 64.22 g to 69.41 g, and the dry weight from 11.69 g to 12.59 g. Combined with salicylic acid, the Fe_3_O_4_ NPs (different concentrations) had a disease incidence of 5.0% to 10.0% and severity of 10.0% to 25.0%. The fresh weight ranged from 51.73 g to 57.78 g, and the dry weight from 7.14 g to 9.07 g. Control group fresh weight: 70.77 g (− control), 6.32 g (+ control); dry weight: 13.10 g (− control), 3.48 g (+ control).

#### 3.3.3. Impact of Various Nanoparticles on the Chlorophyll Content and Phenolic Compounds in Onions Infected by *S. cepivorum*

The Fe_3_O_4_ NPs showed varying impacts on the chlorophyll levels (Table 3). At 1000 ppm, the chlorophyll content was 0.521, chlorophyll b was 0.121, and carotenoids were 0.321. Similar trends were observed at higher concentrations of 1200 ppm and 1400 ppm, with slight increases in the chlorophyll content. The Cu NPs had a minimal impact on the chlorophyll content. Across all the concentrations, there were slight fluctuations in the chlorophyll a, chlorophyll b, and carotenoid levels. The ZnO NPs displayed a significant improvement in the chlorophyll content with increasing concentrations. At 1000 ppm, chl. a was 0.670, chl. b was 0.159, and carotenoids were 0.418. The levels further increased at both the 1200 and 1400 ppm concentrations, indicating a positive effect on chlorophyll production. The combination of salicylic acid with Fe_3_O_4_ NPs also had variable effects on the chlorophyll content. At 1000 ppm, chl. a was 0.572, chl. b was 0.113, and carotenoids were 0.368. The levels increased slightly at 1200 ppm and remained consistent at 1400 ppm. The chlorophyll content was significantly lower in the control group without nanoparticles. Chlorophyll a was 0.199, chl. b was 0.052, and carotenoids were 0.259. Overall, the ZnO NPs demonstrated the most significant positive impact on the chlorophyll content in *S. cepivorum*-infected onions. The Fe_3_O_4_ NPs and the combination of salicylic acid with Fe_3_O_4_ NPs showed moderate effects, while Cu NPs had minimal effects. The control group had the lowest chlorophyll levels, highlighting the detrimental impact of the infection without nanoparticle treatment.

The results in Table 3 also reveal the varying effects of the tested nanoparticles on the phenolic compounds in *S. cepivorum*-infected onion plants. The Fe_3_O_4_ NPs exhibited varied effects on the phenolic levels, with the total phenols at 1000 ppm measuring 6.28, free phenols at 4.81, and related phenols at 2.59. Similar trends were observed at higher concentrations (1200 ppm and 1400 ppm), with fluctuations noted. The Cu NPs had mixed effects, showing slight variations in the total, free, and related phenols across all the concentrations. The ZnO NPs displayed minor fluctuations, with the total phenols at 1000 ppm at 3.60, free phenols at 1.82, and related phenols at 1.73. The combination of salicylic acid with Fe_3_O_4_ NPs also exhibited variable effects, with the total phenols at 1000 ppm measuring 6.70, free phenols at 5.64, and related phenols at 1.23. The control group’s phenolic levels were notably lower.

### 3.4. In Vivo Assessment of Three Chemical Inducers

#### 3.4.1. Assessment of the effect of Three Inducers on the Disease Assessment and Growth Parameters

The results in Table 4 and Figure 2B suggest that SA at concentrations of 250 ppm, 500 ppm, and 1000 ppm showed promising results in reducing the disease incidence and severity. At 250 ppm, the disease incidence was 10.0% and the disease severity was 25.0%. As the concentration increased to 500 ppm and 1000 ppm, the disease incidence remained at 10.0%, while the disease severity decreased to 10.0%. Additionally, the fresh weight and dry weight of the plants increased with higher salicylic acid concentrations, indicating a positive impact on plant growth. Potassium phosphate dibasic (PDP) at concentrations of 1000 ppm, 1200 ppm, and 1400 ppm exhibited mixed effects on the disease assessment and plant growth. The disease incidence remained relatively high at all the concentrations, ranging from 20.0% to 30.0%, while the lowest disease severity was observed at 1200 ppm (30.0%).

The plant growth parameters, including the fresh weight and dry weight, increased with higher PDP concentrations, suggesting a potentially positive effect despite the disease’s presence. Ascorbic acid showed limited efficacy in reducing the disease incidence and severity, with a consistently high incidence (40.0% to 50.0%) and severity (35.0% to 40.0%). Plant growth slightly improved with increasing ascorbic acid concentrations. In summary, SA effectively lowered the disease incidence and severity while promoting plant growth in *S. cepivorum*-infected onions. PDP exhibited mixed effects, with some benefits at higher concentrations. Ascorbic acid had limited efficacy in disease control and minimal impacts on plant growth. The control group had the highest disease incidence and severity and compromised plant growth.

#### 3.4.2. Impact of Three Inducers on the Chlorophyll Content and Phenolic Compounds

The findings highlighted the potential of salicylic acid, PDP, and ascorbic acid to modulate the chlorophyll content and phenolic compounds in *S. cepivorum*-infected onions (Table 5). Salicylic acid, notably at 1000 ppm, consistently showed the highest chlorophyll levels, with significant increases in chlorophyll a, chlorophyll b, and carotenoids. The highest concentration (1400 ppm) of PDP resulted in the highest chlorophyll a and b levels. Ascorbic acid, particularly at 1000 ppm, exhibited the highest chlorophyll values. These concentration-dependent responses emphasize the importance of carefully optimizing the application of these chemical inducers. Conversely, the control group without any chemical treatment consistently displayed the lowest chlorophyll levels.

Additionally, SA at concentrations of 250, 500, and 1000 ppm consistently increased the total phenols, free phenols, and related phenols in infected onions. Particularly at 500 ppm, there was a notable increase in all three phenolic categories, indicating a concentration-dependent effect. For PDP, at 1000 ppm, there was a decrease in the total phenols and related phenols but a slight increase in the free phenols. However, at 1200 ppm and 1400 ppm, there was a substantial increase in all three categories of phenolic compounds. Ascorbic acid, at concentrations of 500, 750, and 1000 ppm, consistently increased the total phenols, free phenols, and related phenols. The highest concentration (1000 ppm) showed the most significant enhancement, emphasizing the positive influence of ascorbic acid on phenolic compound synthesis in onions under pathogenic stress.

### 3.5. The Impact of Various Fungicides on S. cepivorum

#### 3.5.1. The In Vitro Impact of Various Fungicides on the Linear Growth Reduction

The data presented in Table S3 and Figure S6A indicate that Bellis^®^ 38% WG, at concentrations of 1 ppm, 10 ppm, and 25 ppm, demonstrated a significant decrease in the fungal linear growth, with values ranging from 0.5 to 0.7 cm. The reduction percentages were notably high, ranging from 92.22% to 94.44%. Similarly, Topsin M at concentrations of 50 ppm, 100 ppm, and 200 ppm exhibited a notable reduction in the mean linear growth, with the reduction percentages ranging from 90.0% to 94.44%. BOGARD, at concentrations of 1 ppm and 10 ppm, resulted in the complete inhibition of *S. cepivorum* growth, yielding a reduction percentage of 100%. The impact of these fungicides on mycelial malformation was observed under a stereomicroscope (Figure S6B).

#### 3.5.2. The In Vivo Effect of Different Fungicides on the Disease Assessment and Growth Parameters

The data in Table 6 and Figure 2C present the in vivo efficiency of three tested fungicides, indicating the fact that Bellis^®^ 38% WG, at concentrations of 1 ppm, 10 ppm, and 25 ppm, exhibited a substantial decrease in the disease incidence and severity. Notably, at 25 ppm, the disease incidence and severity were completely inhibited. This was accompanied by increased fresh and dry weights, indicating a positive impact on the plant growth parameters. Topsin M, across the concentrations (50 ppm, 100 ppm, and 200 ppm), showed varying degrees of disease reduction, with the highest concentration demonstrating the most substantial decrease. Similar to Bellis, Topsin M contributed to improved plant growth parameters. BOGARD 250 g/L, even at the lowest concentration (1 ppm), completely inhibited the disease incidence and severity while positively influencing the fresh and dry weights.

#### 3.5.3. Impact of Three Fungicides on the Chlorophyll Content and Phenolic Compounds

In the impact assessment of the three fungicides on the chlorophyll content in *S. cepivorum*-infected onions, the highest and lowest values within each treatment revealed notable trends, as indicated in Table 7. In the Bellis^®^ 38% WG treatment, the chl. a, chl. b, and carotenoid levels remained relatively stable across the concentrations (1 ppm, 10 ppm, and 25 ppm), with no distinct highest or lowest values observed. For Topsin M (70% WP), the highest chlorophyll levels were recorded at 50 ppm. At the same time, the lowest values were consistently observed at 100 ppm and 200 ppm, indicating a concentration-dependent decrease in the chlorophyll content. In the BOGARD 250 g/L treatment, the highest chlorophyll levels were generally found at 10 ppm, and the lowest values were observed at 25 ppm, suggesting a concentration-dependent impact on the chlorophyll content.

In addition, the impact of the tested fungicides on the phenolic compounds demonstrated that the concentrations of 1 ppm, 10 ppm, and 25 ppm for each fungicide exhibited a consistent trend of increased phenolic compounds compared to the control groups (+ and −). Bellis, Topsin M, and BOGARD exhibited a dose-dependent rise in the total, free, and related phenols. The highest total phenols were observed in Topsin M at 200 ppm, with a value of 4.74, whereas the lowest total phenols were recorded in Bellis at 1 ppm, with a value of 4.30. At the same time, the highest free phenols were observed in BOGARD at 25 ppm, with a value of 4.12, while the lowest free phenols were found in Topsin M at 50 ppm, with a value of 3.50. In addition, the highest related phenols were observed in Bellis and Topsin M at 10 ppm, both with values of 0.75, whereas the lowest related phenols were recorded in BOGARD at 10 ppm, with a value of 0.32. The positive control (+) displayed lower phenolic levels, while the negative control (−) exhibited elevated phenolics.

## 4. Discussion

Nanomaterials have potent antimicrobial effects due to the increased surface contact with cells, triggering reactions that generate reactive oxygen species (ROS), damaging proteins, and disrupting cell membranes. These interactions make nanomaterial-based antimicrobials resistant to pathogen resistance [32,33,34,35].

In addition, nanoparticles can stimulate morphological and physiological changes in plants, with their efficacy influenced by factors like the composition, size, surface properties, reactivity, and dosage [9]. The concentration of NPs is crucial for their effectiveness, and nanotechnology presents unique applications in biotechnology and agriculture [10]. NPs can serve as targeted carriers, delivering substances like herbicides, pesticides, fertilizers, or genes to specific cellular organelles in plants [11]. Despite the potential benefits, inadequate information exists on NP toxicity in plants, and few studies have explored the mechanisms underlying NP influences on plant growth. Understanding NP action’s physiological, biochemical, and molecular mechanisms is essential for optimizing the impact on host growth [12].

It is evident that the pathogenicity of *S. cepivorum* isolates varies significantly, impacting the Giza 20 onion cultivar under controlled greenhouse conditions. The maximum disease incidence and severity were observed in the third isolate from Abu-Hamad, suggesting a potent pathogenic strain in that region. This aligns with previous research by Tyson et al. [36], who found regional variations in the virulence of *S. cepivorum* isolates. The diverse responses of isolates one and two, from Zagazig and Minia Elkamh, may be due to environmental factors or genetic differences in the isolates themselves [37].

The reduced plant weights, both fresh and dry, further underscore the detrimental effects of the pathogen on the Giza 20 onion cultivar. Similar findings were reported by Elshahawy et al. [38], who demonstrated a correlation between the disease severity and diminished plant growth in onion crops infected with *S. cepivorum*. Isolate four from Abu-Kabeer exhibited intermediate effects, indicating a moderate pathogenicity compared to the other isolates. This aligns with the concept of strain-specific variations in virulence, as discussed by Garcia-Rubio et al. [39]. Several factors may contribute to the observed trends, such as the genetic diversity of *S. cepivorum* isolates [40,41] and environmental conditions influencing the pathogen–host interaction [42]. Understanding these factors is crucial for developing effective disease management strategies.

The effect of diverse NPs on the linear growth and reduction percentage of *S. cepivorum* provides insights into potential antifungal strategies [43]. The findings align with previous studies on the antifungal properties of nanoparticles. For instance, research by Wu et al. [44] on Fe_3_O_4_ NPs corroborates the dose-dependent inhibition observed in this study. The substantial reduction in linear growth with increasing concentrations of Fe_3_O_4_ NPs suggests a potential mechanism of action, possibly involving interference with fungal cell membranes or vital cellular processes, as discussed by Rawat et al. [45]. The Cu NPs and ZnO NPs also exhibited dose-dependent inhibitory effects, consistent with studies on their antifungal activities against various pathogens [46]. The high reduction percentages at 100 μg/L indicate their potential as effective agents against *S. cepivorum*, which aligns with Derbalah et al. [47]. These nanoparticles’ mode of action could involve ROS generation, disrupting cellular functions [48].

Combining salicylic acid with Fe_3_O_4_ NPs demonstrated remarkable efficacy, achieving a substantial reduction percentage [49]. This aligns with studies highlighting the synergistic effects of combining nanoparticles with other antifungal agents [50]. Salicylic acid, known for its role in plant defense mechanisms, may enhance the antifungal activity of nanoparticles through various biochemical pathways [49]. Factors such as the nanoparticle size, surface charge, and interactions with fungal cell components may influence their efficacy [51]. Iron acts as a cofactor for various enzyme systems and plays a crucial role in plant cells, constituting a significant portion of metal microelements and supporting the activity of enzymes like peroxidases and catalases [52].

The results showed that the Fe_3_O_4_ NPs at varying concentrations resulted in disease reduction and improved plant growth, while the Cu NPs also exhibited disease reduction and increased plant growth. Additionally, the ZnO NPs showed similar effects. The Fe_3_O_4_ NPs further reduced disease and improved plant growth when combined with salicylic acid. These results suggest the ability of these nanoparticles to manage *S. cepivorum* infection in onions and growth enhancement, which is in line with Faizan et al. [53]. A study by Khan et al. [54] found that Fe_3_O_4_ NPs efficiently lowered the disease incidence and severity in tomato plants infected with a fungal pathogen. Similarly, the work of Gaba et al. [55] demonstrated the disease-suppressive effects of Cu nanoparticles on Alternaria blight disease and other various plant pathogens, as reported by Elmer and White [56]. Furthermore, the study by Zhang et al. [57] highlighted the positive influence of ZnO NPs on rice growth and disease resistance.

The trends observed in our study could be because of the unique physicochemical properties of the nanoparticles. For example, Fe_3_O_4_ NPs were confirmed to induce systemic resistance in plants by modulating defense-related gene expression and activating signaling pathways [58]. Similarly, Cu NPs have been shown to possess antifungal properties, disrupting the cell membranes of pathogens and inhibiting their growth [59]. Moreover, ZnO NPs enhanced host growth by promoting nutrient uptake and modifying physiological processes [60,61]. Moreover, the combination of salicylic acid and Fe_3_O_4_ NPs resulted in further disease reduction and improved plant growth [62,63], demonstrating nanoparticles’ enhanced antifungal activity when combined with plant defense signaling compounds. ZnO NPs had the most significant positive impact, increasing the chlorophyll levels at higher concentrations [64,65].

This study demonstrated that different nanoparticles exhibit varying effects on the disease incidence and severity in *S. cepivorum*-infected onions. The Cu NPs and the combination of salicylic acid and Fe_3_O_4_ NPs showed promising results in reducing the disease incidence and severity. This aligns with the findings of a study by Pariona et al. [66], which reported the antifungal activity of copper nanoparticles against plant pathogens. The ability of the Cu NPs to suppress the growth and development of fungal pathogens could explain their effectiveness in reducing the disease incidence and severity in infected onions. Therefore, Cu NPs are an affordable solution for combating fungal diseases, showing strong antifungal activity. Studies reveal that Cu NPs can penetrate cell membranes, causing rupture, impeding colony growth, and inhibiting mycelium and spore growth [67]. The spherical shape enhances their antifungal activity [68]. Interestingly, Cu NPs completely inhibited phytopathogens at a concentration of 1000 ppm, indicating their promise in managing agricultural diseases [69]. CuO-NPs possess versatile properties, making them valuable in drug delivery and anticancer, antifungal, antioxidant, and antibacterial applications [70,71].

Cu NPs can modify physiological and biochemical processes in plants, impacting germination and growth. Elevated concentrations in the solution culture have been detrimental to wheat plants, whereas lower concentrations in the growth medium improved the yield parameters [72]. Despite copper being an essential micronutrient, excessive exposure can lead to phytotoxicity, inducing oxidative stress and DNA damage in plants [73]. CuO-NPs and ZnO-NPs showed stronger antifungal effects, forming larger inhibition zones and causing the distortion of fungal hyphae, inhibiting conidia and conidiophores’ expansion, leading to cell death [71,74].

The application of zinc oxide (ZnO) and copper (Cu) nanoparticles has demonstrated improvements in seed quality [75]. This treatment led to increased growth parameters, relative water content, and biochemical factors like total phenolic and proline contents, catalase (CAT), peroxidase (POD), and glutathione S-transferase [76]. Similarly, treating chickpea plants with ZnO NPs below 30 nm at a concentration of 1.5 ppm significantly increased the total biomass compared to 10 ppm. Higher concentrations (10 ppm) were correlated with decreased relative water levels in plant leaves [77]. Nanoparticles were used as nano-priming agents for antibacterial and antifungal activities against phytopathogens, besides enhancing plant growth [78]. Using Fe_2_O_3_ NPs as co-fertilizers at lower concentrations can effectively improve chickpea growth [79]. Studies by George et al. [80] on the anti-fungal activity of ZnO and TiO_2_ nanoparticles against various fungal pathogens demonstrated their efficacy compared to bulk particles. Both nanoparticles exhibited high antimicrobial activities against bacteria and phytopathogenic fungi. ZnO NPs displayed better anti-fungal properties than TiO_2_. The most effective nanoparticles for antimicrobial properties typically have diameters in the range of 10–100 nm. Both ZnO NPs and CuO NPs have shown broad-spectrum antimicrobial activities, making them promising candidates for various applications, including antiviral, anticancer, and UV-protection [81,82]. ZnO-NPs have diverse applications, including sensors, drug delivery, wastewater treatment, tissue engineering, catalysts, and antimicrobials. Sustainable synthesis methods are crucial for widespread use [83].

In terms of the phenolic compounds, the Fe_3_O_4_ NPs caused variable effects on the phenolic compounds in *S. cepivorum*-infected onions, with concentrations of 1000 ppm, 1200 ppm, and 1400 ppm leading to fluctuations in the total, free, and related phenols. Cai et al. [84] found that Fe_3_O_4_ NPs, when foliar exposed to *Nicotiana benthamiana*, demonstrate evidence of nanoparticle uptake, acting as both plant growth promoters and elicitors of defense responses against plant viruses. Also, Fe_3_O_4_ NPs have increased phenolic compound levels [63]. The Cu NPs and ZnO NPs minimally impacted the phenolic compound levels in infected onions, aligning with studies on *Hypericum perforatum* L. using various metal oxide nanoparticles [85]. The combination of salicylic acid and Fe_3_O_4_ NPs yielded concentration-dependent effects on the phenolic compounds, reflecting the complex interactions between salicylic acid, nanoparticles, and the plant–pathogen system. The control group, representing infected and uninfected onions without nanoparticle treatment, exhibited the lowest phenolic compound levels, indicating that *S. cepivorum* infection negatively influences their synthesis or accumulation due to plant–pathogen interactions [86].

The three tested inducers, salicylic acid, PDP, and ascorbic acid, exhibited potential impacts on disease management and plant growth. Salicylic acid, at concentrations of 250 ppm, 500 ppm, and 1000 ppm, demonstrated promising results in decreasing both the disease incidence and severity. These findings align with previous studies highlighting salicylic acid’s role in stimulating plant defensive mechanisms versus various pathogens [87,88]. Salicylic acid is known to activate systemic acquired resistance (SAR) in plants, which enhances their ability to ward off infections [88]. The increase in the salicylic acid concentration resulted in a decline in the disease severity, indicating a concentration-dependent response, in line with Thaler et al. [89]. Salicylic acid is fundamental in regulating plant defense responses [90]. The positive impact of salicylic acid on plant growth, as indicated by the increased fresh and dry weights, is consistent with studies demonstrating its involvement in promoting overall plant health and development [91].

The application of SA has shown positive effects on the growth vigor of both root and shoot [92]. This improvement is attributed to hormonal regulation, increased leaf turgidity through stomatal closure, reduced transpiration rates, and enhanced relative water content [93]. As a phenolic phytohormone, SA acts as a systemic signal that triggers the local systemic resistance response. It serves as a key regulator in the signaling network for plants facing abiotic and biotic stresses, leading to the increased activity of the PAL, PO, and PPO enzymes, along with elevated phenolic contents. The exogenous supplementation of SA stimulates systemic resistance and enhances plant performance under both abiotic and biotic stress [94,95,96].

PDP exhibited mixed effects on the disease severity and plant growth. This variation in response might be due to the complex interaction between phosphorus, potassium, and disease resistance in plants [97]. Potassium is known to enhance plant resistance toward diverse phytopathogens [98,99]. The positive impact of higher PDP concentrations on the plant growth parameters, despite the presence of the disease, is consistent with studies indicating the role of phosphorus and potassium in enhancing plant vigor and tolerance to stress [100]. However, the mixed effects on the disease assessment suggest that PDP may have a more nuanced impact on the specific pathogen–host interaction under consideration.

Ascorbic acid (AsA) demonstrated limited efficacy in reducing the disease incidence and severity. The consistently high disease incidence and severity, even at higher concentrations of ascorbic acid, indicate that it may not be as effective in managing onion white rot disease. However, ascorbic acid has demonstrated effectiveness in minimizing conidia numbers’ impact and powdery mildew severity on flower clusters and fruit set [101]. Similar findings have been reported in studies on other plant–pathogen systems, where the effectiveness of ascorbic acid as a disease control agent varies [102,103]. The slight improvements in the plant growth parameters with increasing ascorbic acid concentrations may suggest a potential role in promoting plant health. Still, the limited impact on disease control raises questions about its efficacy as a standalone control measure for this specific disease [104,105,106,107].

Ascorbic acid is a crucial plant antioxidant, acting as a frontline defense against ROS induced by environmental factors like wounding, ozone, high salinity, and pathogens [105,107]. Its role extends to plant–pathogen interactions, contributing to the overall plant growth, development, and stress tolerance. As part of a complex defense network, AsA, along with glutathione and enzymatic antioxidants, maintains a redox environment essential for regulating defense pathways. These pathways involve the activation of the NPR1 regulatory transcription factor, expression of defense genes, cell wall reinforcement, and modulation of defense–hormonal signaling networks. AsA also acts as an inducer or part of the induced resistance processes triggered by elicitors like β-aminobutyric acid, jasmonic acid, methyljasmonate, and extracellular polysaccharides [104,105,106,107].

The varying effects of PDP on the phenolic compounds at different concentrations highlight the complex interplay between nutrient availability and plant defense responses. The decrease in the total phenols and related phenols at 1000 ppm may be attributed to the intricate balance between phosphorus and potassium, where an excess of one nutrient may affect the plant’s ability to mount an effective defense [108]. Moreover, the consistent increase in the phenolic compounds with ascorbic acid supplementation aligns with the antioxidant properties of ascorbic acid, which can stimulate the synthesis of secondary metabolites, including phenolics [109]. The elevated levels of the phenolic compounds, particularly at 1000 ppm, suggest that ascorbic acid positively influences the induction of plant defense mechanisms against *S. cepivorum*.

In turn, the results highlight the effectiveness of Bellis, Topsin M, and BOGARD in inhibiting the linear growth of *S. cepivorum*. This aligns with the existing literature highlighting the efficacy of Bellis^®^ in controlling fungal pathogens [110,111,112]. Similarly, Topsin M demonstrated potent antifungal activity against various pathogens [113,114]. Moreover, other findings have been reported, emphasizing BOGARD’s strong antifungal properties [115]. Fungicides, such as those tested in this study, are known to interfere with crucial cellular processes in fungi, including cell wall synthesis and energy production [112,114]. The observed effectiveness of the tested fungicides in inhibiting the linear growth of *S. cepivorum* can be attributed to their distinct active ingredients and modes of action [116]. Bellis^®^ contains a combination of pyraclostrobin and boscalid. Pyraclostrobin is a strobilurin fungicide that disrupts fungal respiration by inhibiting the electron transport chain in mitochondria, decreasing ATP production [117,118]. Conversely, boscalid acts on the succinate dehydrogenase enzyme, disrupting the tricarboxylic acid cycle in fungi [118,119]. The dual action of pyraclostrobin and boscalid disrupts multiple cellular processes in the fungus, resulting in a potent antifungal effect [120]. This combination likely contributes to the high reduction percentages observed in the linear growth of *S. cepivorum*. Topsin M contains thiophanate methyl, which belongs to the benzimidazole class of fungicides. Thiophanate methyl inhibits microtubule assembly in fungi, disrupting cell division and growth [121,122]. The observed reduction in the linear growth of *S. cepivorum* can be attributed to the disruption of cell division processes by thiophanate methyl [123]. The fungicidal activity of this benzimidazole likely hampers the pathogen’s ability to proliferate and spread [124]. As for the BOGARD, it contains tebuconazole, a triazole fungicide. Triazoles interfere with the process of producing ergosterol, which is necessary for the membranes of fungi, leading to membrane destabilization and cell death [125]. The inhibition of ergosterol biosynthesis disrupts the integrity of the fungal cell membrane, compromising its structural integrity and function [126]. This likely contributes to the complete growth inhibition observed at lower concentrations of BOGARD. Understanding the specific modes of action of these fungicides provides insights into their effectiveness against *S. cepivorum*. The combination of multiple active ingredients in Bellis^®^, the microtubule disruption by the thiophanate methyl in Topsin M, and the membrane-targeting action of the tebuconazole in BOGARD collectively contribute to the potent antifungal effects observed in the study.

This study offers insightful information on the complex reactions of onion plants to different fungicides and concentrations. While Bellis^®^ demonstrated stability in the chlorophyll content, Topsin M and BOGARD showed concentration-dependent trends. These findings highlight the importance of optimizing the fungicide concentrations for effective disease management while minimizing potential physiological stress in plants. The chlorophyll levels in Bellis^®^ treatments suggest that it exerts minimal direct stress on the photosynthetic apparatus. Similar findings have been reported in studies assessing the impact of fungicides on the chlorophyll content of various crops [112,127]. The absence of a clear concentration-dependent response might indicate that it primarily functions as a disease control agent without causing significant physiological disruptions. Also, some studies indicated that certain fungicides, including those containing thiophanate methyl, as in Topsin M, can induce stress in the photosynthetic apparatus at higher concentrations [128,129]. The decrease in the chlorophyll levels may be attributed to the fungicide’s impact on cellular processes involved in photosynthesis, emphasizing the importance of optimizing fungicide concentrations to avoid adverse effects on plant health [129]. The observed variability suggests that while lower concentrations may have a minimal impact on chlorophyll, higher concentrations can lead to physiological stress. This emphasizes the importance of carefully calibrating the fungicide doses for optimal disease control and minimal impact on plant physiology [130].

In addition, the consistent increase in phenols across all the fungicide treatments suggests a systemic induction of phenolic compounds, indicative of an enhanced defense response against the pathogen [131]. Notably, the tested fungicides demonstrated a dose-dependent increase in the phenolic compounds, emphasizing their potential to induce the synthesis of secondary metabolites involved in plant defense mechanisms. The control groups exhibited significantly lower phenolic levels, with the positive control (+) indicating the detrimental impact of the pathogen and the negative control (−) highlighting the inherent baseline stress response induced by the plant immune system [132]. This consistent increase in the phenolic compounds aligns with the current understanding of plant defense responses, where the activation of secondary metabolites, including phenolics, is a common strategy to combat pathogenic attacks [133]. The findings suggest that the tested fungicides contribute to disease management and induce a systemic defense response in the form of increased phenolic compounds, potentially fortifying the plants against further pathogenic infection.

## 5. Conclusions

This research elucidates the potential of nanoparticles and chemical inducers as sustainable alternatives for managing onion white rot caused by *S. cepivorum*. The variability in pathogenicity among *S. cepivorum* isolates emphasizes the need for adapted approaches to disease management. Fe_3_O_4_ NPs, Cu NPs, and ZnO NPs exhibit concentration-dependent inhibition of *S. cepivorum* growth, showcasing their antifungal properties. Salicylic acid emerges as a key player, triggering the plant’s immune response and promoting resilience against the pathogen. Traditional fungicides, such as Bellis^®^ 38% WG, Topsin M, and BOGARD, demonstrate potent inhibitory effects, providing effective options for disease control. The impact of these strategies on the chlorophyll content and phenolic compounds highlights their potential physiological effects on onions. Overall, Fe_3_O_4_, Cu, and ZnO nanoparticles inhibit mycelial growth in vitro, with salicylic acid and Fe_3_O_4_ nanoparticles exhibiting synergistic effects. In vivo, Fe_3_O_4_ nanoparticles at 1000–1400 ppm significantly reduce the disease incidence and severity. ZnO nanoparticles enhance the chlorophyll content, while Cu nanoparticles have minimal effects. Additionally, salicylic acid reduces the disease incidence and severity, promotes growth, and enhances the chlorophyll values and phenolic compounds. This research contributes valuable insights to the development of eco-friendly and sustainable approaches for onion white rot management, paving the way for the more harmonious coexistence of agriculture and the environment.

## Figures and Tables

**Table 1:** Evaluation of the pathogenicity of the causative agent of white rot disease on the Giza 20 onion cultivar conducted under controlled greenhouse conditions.

Treatment	Place of Isolates	Disease Assessment		Plant Growth Parameter	
		Disease Incidence %	Disease Severity %	Fresh Weight (g)	Dry Weight (g)
*S. cepivorum* isolate 1	Zagazig	60.00 b	87.00 b	12.85 b	4.26 b
*S. cepivorum* isolate 2	Minia Elkamh	60.00 b	83.00 b	12.36 b	4.58 b
*S. cepivorum* isolate 3	Abu-Hamad	80.00 a	100.00 a	6.32 c	3.48 b
*S. cepivorum* isolate 4	Abu-Kabeer	65.00 b	85.00 b	10.53 b	4.12 b
Control		0.00 c	0.00 c	70.77 a	13.10 a
L.S.D.		9.414	8.314	4.052	1.354

The assigned letters (‘a’, ‘b’, ‘c’) represent Duncan’s multiple range test outcomes at *p* < 0.05 significance. Different letters signify significant treatment differences, while similar ones denote non significance.

**Table 2:** Impact of diverse nanoparticles on the disease assessment and plant growth parameters in *S. cepivorum*-infected onions.

Treatment	Concentration	Disease Assessment		Plant Growth Parameter	
		Disease Incidence %	Disease Severity %	Fresh Weight (g)	Dry Weight (g)
Fe_3_O_4_ NPs	1000 ppm	80.00 a	90.00 ab	36.60 j	3.85 d
	1200 ppm	70.00 a	90.00 ab	37.11 ij	4.20 d
	1400 ppm	65.00 a	80.00 b	37.85 hij	4.85 d
Cu NPs	1000 ppm	25.00 bc	15.00 ef	49.31 ef	7.05 c
	1200 ppm	15.00 bcd	10.00 fg	50.45 ef	7.28 bc
	1400 ppm	5.00 d	10.00 fg	50.98 e	7.25 bc
ZnO NPs	1000 ppm	30.00 b	40.00 c	64.22 b	11.69 a
	1200 ppm	30.00 b	30.00 cd	69.12 ab	12.42 a
	1400 ppm	25.00 bc	25.00 de	69.41 ab	12.59 a
Salicylic acid with Fe_3_O_4_ NPs	1000 ppm	10.00 cd	25.00 de	51.73 de	7.14 c
	1200 ppm	5.00 d	10.00 fg	56.86 cd	9.25 b
	1400 ppm	5.00 d	10.00 fg	57.78 c	9.07 bc
Control	+	80.00 a	100.00 a	6.32 k	3.48 d
	−	0.00 d	0.00 g	70.77 a	13.10 a
L.S.D.	Nano	7.593	5.510	2.098	0.807
	concentration	5.559	4.034	1.536	0.591
	Nano × concentration	19.714	14.306	5.447	2.095

+ Onions infected with *S. cepivorum* without nanoparticle treatments; − Negative control: Uninfected onions treated with nanoparticles. The assigned letters (‘a’, ‘b’, ‘c’, ‘d’, ‘e’, ‘f’, ‘g’, ‘h’, ‘i’, ‘j’, ‘k’) represent Duncan’s multiple range test outcomes at *p* < 0.05 significance. ‘×’ indicates variable interaction.

**Table 3:** Impact of diverse nanoparticles on the chlorophyll content and phenolic compounds in onions infected by *S. cepivorum*.

Treatment	Concentration	Chlorophyll			Phenols		
		Chl. a	Chl. b	Carotenoids	Total Phenols	Free Phenols	Related Phenols
Fe_3_O_4_ NPs	1000 ppm	0.521 f	0.121 d	0.321 g	6.28 a	4.81 b	2.59 b
	1200 ppm	0.526 f	0.118 d	0.334 fg	4.47d	3.44 d	2.64 b
	1400 ppm	0.679 c	0.238 a	0.417 c	5.65 b	4.27 c	2.48 b
Cu NPs	1000 ppm	0.524 f	0.113 de	0.335 fg	5.58 b	1.81 ef	2.19 bcd
	1200 ppm	0.558 d	0.113 d	0.338 efg	5.41 b	1.63 f	2.21 bcd
	1400 ppm	0.564 d	0.114 d	0.356 def	4.51 b	1.87 ef	2.30 abc
ZnO NPs	1000 ppm	0.670 c	0.159 c	0.418 c	3.60 e	1.82 ef	1.73 defg
	1200 ppm	0.751 b	0.1829 b	0.459 b	3.83 e	1.88 ef	1.85 cdef
	1400 ppm	0.829 a	0.242 a	0.520 a	4.87 cd	2.22 e	2.83 a
Salicylic acid with Fe_3_O_4_ NPs	1000 ppm	0.572 d	0.113 d	0.368 de	6.70 a	5.64 a	1.23 gh
	1200 ppm	0.674 c	0.250 a	0.419 c	5.26 bc	4.46 bc	1.09 h
	1400 ppm	0.677 c	0.241 a	0.420 c	5.21 bc	4.28 c	1.29 fgh
Control	+	0.199 j	0.052 g	0.259 h	1.41 g	1.61 f	0.05 i
	−	0.429 h	0.099 e	0.246 h	2.50 f	2.16 e	2.11 bcde
L.S.D.	Nano	0.409	0.277	0.012	0.191	0.186	0.217
	concentration	0.960	0.863	0.872	0.140	0.136	0.159
	Nano × concentration	0.014	0.014	0.032	0.497	0.482	0.562

+ Onions infected with *S. cepivorum* without nanoparticle treatments; − Negative control: Uninfected onions treated with nanoparticles. The letters (‘a’, ‘b’, ‘c’, ‘d’, ‘e’, ‘f’, ‘g’, ‘h’, ‘i’, ‘j’) represent Duncan’s multiple range test outcomes at *p* < 0.05 significance. Different letters signify significant treatment differences, while similar ones denote nonsignificance. ‘×’ indicates variable interaction.

**Table 4:** In vivo assessment of the effect of three chemical inducers on the disease assessment and growth parameters in onion white rot disease caused by *S. cepivorum*.

Treatment	Concentration	Disease Assessment		Plant Growth Parameter	
		Disease Incidence %	Disease Severity %	Fresh Weight (g)	Dry Weight (g)
Salicylic acid	250 ppm	10.00 f	25.00 e	52.53 h	7.54 f
	500 ppm	10.00 f	10.00 f	55.75 g	9.62 e
	1000 ppm	5.00 fg	10.00 f	57.21 f	9.28 e
Potassium dibasic phosphate (PDP)	1000 ppm	30.00 d	35.00 cd	60.07 e	10.84 d
	1200 ppm	30.00 d	30.00 de	64.13 d	11.83 c
	1400 ppm	20.00 e	32.33 d	63.76 d	12.26 b
Ascorbic acid	500 ppm	50.00 b	40.00 c	69.91 c	11.64 c
	750 ppm	40.00 c	35.00 cd	71.12 ab	12.38 b
	1000 ppm	40.00 c	35.00 cd	72.24 a	12.28 b
Control	+	80.00 a	100.00 a	19.12 j	3.50 i
	−	0 g	0 g	70.79 bc	13.13 a
L.S.D.	Inducer	4.998	3.640	0.568	0.175
	concentration	4.471	3.256	0.508	0.156
	Inducer × concentration	9.100	7.280	1.136	0.350

+ Positive control: Onions infected with *S. cepivorum* without chemical inducers treatments. − Negative control: Uninfected onions treated with chemical inducers. The letters (‘a’, ‘b’, ‘c’, ‘d’, ‘e’, ‘f’, ‘g’, ‘h’, ‘i’, ‘j’) represent Duncan’s multiple range test outcomes at *p* < 0.05 significance. Different letters signify significant treatment differences, while similar ones denote non-significance. ‘×’ indicates variable interaction.

**Table 5:** Impact of three chemical inducers on the chlorophyll content and phenolic compounds in onions infected by *S. cepivorum*.

Treatment	Concentration	Chlorophyll			Phenols		
		Chl. a	Chl. b	Carotenoids	Total Phenols	Free Phenols	Related Phenols
Salicylic acid	250 ppm	0.652 d	0.253 d	0.255 e	4.58 c	3.72 b	1.60 fg
	500 ppm	0.642 d	0.241 de	0.418 c	5.26 b	4.32 a	2.37 b
	1000 ppm	0.678 c	0.741 b	0.421 c	5.24 b	4.50 a	2.36 b
Potassium dibasic phosphate (PDP)	1000 ppm	0.523 f	0.107 i	0.354 d	2.33 fg	1.86 de	2.17 cd
	1200 ppm	0.568 e	0.233 ef	0.353 d	5.37 b	1.65 de	2.17 cd
	1400 ppm	0.579 e	0.702 c	0.480 a	5.35 b	1.66 de	2.33 bc
Ascorbic acid	500 ppm	0.703 b	0.233 ef	0.433 c	3.46 d	1.93 cd	1.71 ef
	750 ppm	0.739 a	0.221 f	0.456 b	4.75 c	3.70 b	2.16 cd
	1000 ppm	0.750 a	0.769 a	0.490 a	5.69 a	4.27 a	2.90 a
Control	+	0.198 h	0.049 k	0.257 e	1.41 h	1.61 e	0.05 h
	−	0.417 g	0.086 j	0.247 ef	2.50 fg	2.16 c	2.11 d
L.S.D.	Inducer	0.561	0.304	0.247	0.125	0.149	0.092
	concentration	0.763	0.533	0.376	0.112	0.134	0.082
	Inducer × concentration	0.015	0.015	0.017	0.250	0.299	0.184

+ Onions infected with *S. cepivorum* without chemical inducers treatments. − Negative control: Uninfected onions treated with chemical inducers. The letters (‘a’, ‘b’, ‘c’, ‘d’, ‘e’, ‘f’, ‘g’, ‘h’, ‘i’, ‘j’, ‘k’) represent Duncan’s multiple range test outcomes at *p* < 0.05 significance. Different letters signify significant treatment differences, while similar ones denote non-significance. ‘×’ indicates variable interaction.

**Table 6:** In vivo efficiency of three fungicides on the disease assessment and growth parameters on onion white rot disease caused by *S. cepivorum*.

Treatment	Concentration	Disease Assessment		Plant Growth Parameter	
		Disease Incidence (%)	Disease Severity (%)	Fresh Weight (g)	Dry Weight (g)
Bellis^®^ 38% WG	1 ppm	5.00 cd	10.00 c	60.02 e	7.20 f
	10 ppm	5.00 cd	10.00 c	62.75 d	11.31 c
	25 ppm	0.00 d	0.00 d	63.52 cd	12.65 ab
Topsin M, 70% WP	50 ppm	20.00 b	15.00 b	60.81 b	10.12 d
	100 ppm	10.00 c	10.00 c	63.38 d	11.60 c
	200 ppm	5.00 cd	10.00 c	64.83 b	12.32 b
BOGARD 250 g/L	1 ppm	0.00 d	0.00 d	63.68 bcd	8.39 e
	10 ppm	0.00 d	0.00 d	64.77 b	12.43 b
	25 ppm	0.00 d	0.00 d	64.69 bc	12.42 b
Control	+	80.00 a	100 a	80.00 a	3.39 g
	−	0.00 d	0.00 d	70.26 a	12.94 a
L.S.D.	Fungicide	4.420	2.210	0.685	0.204
	concentration	3.424	1.712	0.530	0.158
	Fungicide × concentration	7.656	3.828	1.186	0.354

+ Onions infected with *S. cepivorum* without nanoparticle treatments; − Negative control: Uninfected onions treated with nanoparticles. The letters (‘a’, ‘b’, ‘c’, ‘d’, ‘e’, ‘f’, ‘g’) represent Duncan’s multiple range test outcomes at *p* < 0.05 significance. Different letters signify significant treatment differences, while similar ones denote non-significance. ‘×’ indicates variable interaction.

**Table 7:** Impact of three fungicides on the chlorophyll content and phenolic compounds in onions infected by *S. cepivorum*.

Treatment	Concentration	Chlorophyll			Phenols		
		Chlorophyll a	Chlorophyll b	Carotenoids	Total Phenols	Free Phenols	Related Phenols
Bellis^®^ 38% WG	1 ppm	0.571 bc	0.136 a	0.376 b	4.30 cd	3.49 ef	0.72 b
	10 ppm	0.574 bc	0.132 a	0.378 b	4.51 b	3.41 f	0.75 b
	25 ppm	0.572 bc	0.139 a	0.373 b	4.52 b	3.85 bc	0.74 b
Topsin M, 70% WP	50 ppm	0.555 d	0.118 b	0.321 c	4.22 cd	3.50 def	0.70 b
	100 ppm	0.530 f	0.118 b	0.317 c	4.20 cd	3.72 bcd	0.72 b
	200 ppm	0.540 e	0.116 b	0.317 c	4.74 a	3.77 bc	0.72 b
BOGARD 250 g/L	1 ppm	0.564 cd	0.114 b	0.357 bc	4.22 cd	3.64 cde	0.35 d
	10 ppm	0.591 a	0.115 b	0.359 bc	4.17 d	3.93 ab	0.32 d
	25 ppm	0.579 b	0.118 b	3.712 a	4.34 c	4.12 a	0.42 c
Control	+	0.194 h	0.062 d	0.250 d	1.40 f	1.35 h	0.05 e
	−	0.421 g	0.094 c	0.249 d	2.46 e	2.21 j	2.14 a
L.S.D.	Fungicide	0.891	0.689	0.026	0.083	0.129	0.032
	concentration	0.563	0.632	0.020	0.064	0.100	0.025
	Fungicide × concentration	0.0102	0.122	0.044	0.143	0.224	0.055

+ Onions infected with *Sclerotium cepivorum* without nanoparticle treatments; − Negative control: Uninfected onions treated with nanoparticles. The letters (‘a’, ‘b’, ‘c’, ‘d’, ‘e’, ‘f’, ‘g’, ‘h’, ‘j’) represent Duncan’s multiple range test outcomes at *p* < 0.05 significance. Different letters mean significant treatment differences, while similar ones refer to non-significance. ‘×’ indicates variable interaction.

## Data Availability

The original data presented in the study are openly available at https://figshare.com/10.6084/m9.figshare.25444723, accessed on 20 March 2024.

## Supplementary Materials

The following supporting information can be downloaded at: https://www.mdpi.com/article/10.3390/biology13040219/s1, Table S1: List of diverse fungicides containing various chemical active ingredients tested for their efficacy in controlling *S. cepivorum*; Table S2: Influence of diverse nanoparticles utilizing the poisoned media technique on the reduction percentage and linear growth of *S. cepivorum*. Table S3: Effect of different fungicide using poisoned media technique on the reduction percent of the linear growth of *S. cepivorum*; Figure S1: Characterization of Cu NPs: (a) TEM image (b) Particle size analysis (c) Zeta potential analysis (d) XRD pattern of Cu NPs; Figure S2: Characterization of Zn NPs: (a) TEM image (b) Particle size analysis (c) Zeta potential analysis (d) XRD pattern of Zn NPs; Figure S3: Characterization of Fe_3_O_4_ NPs: (a) TEM image (b) Particle size analysis (c) Zeta potential analysis (d) XRD pattern of Fe_3_O_4_ NPs; Figure S4: Characterization of SA-Fe NPs: (a) TEM image (b) Zeta potential analysis of SA (c) Zeta potential of SA-Fe NPs; Figure S5: In vitro evaluation: (A) the application of nanoparticles exerted control, inducing malformation in the morphology of mycelial growth, and preventing the formation of sclerotia. (B) The impact of nanoparticles on mycelial malformation was observed under a light microscope. The mycelial malformation induced by nanoparticles was examined using stereomicroscopy; Figure S6: (A) In vitro application of fungicides. Complete inhibition of mycelium and sclerotia was observed. (B) Examination under a stereomicroscope revealed the effects of fungicide treatment on mycelial formation and sclerotia.
