# Supplementary material for: Nanoparticles and Chemical Inducers: A Sustainable Shield against Onion White Rot
Source: Biology (Basel). 2024 Mar 27;13(4):219. doi: 10.3390/biology13040219 (PMC11048201; doi:10.3390/biology13040219)
Supplement: Supplementary file 1 [file biology-13-00219-s001.zip › biology-2884641-supplementary.pdf]

Supplementary Tables

Table S1. List of diverse fungicides containing various chemical active ingredients tested for their efficacy in controlling *S. cepivorum.*

Fungicide trade name	Active ingredient
Bellis® 38% WG	Pyraclostrobin + Boscalid 38% WG
Topsin M, 70 %WP	Thiophanate methyl 70 %WP
BOGARD 250 g/l	Teubconazole 250 g/l

Table S2. Influence of diverse nanoparticles utilizing the poisoned media technique on the reduction percentage and linear growth of *S. cepivorum*.

Treatment	Concentration	Linear growth mean (cm)	Reduction percent (%)
Fe_3O_4 NPs	25 µg/l	8.50b	5.56g
	50 µg/l	7.70cd	14.44ef
	100 µg/l	7.30de	18.89de
Cu NPs	25 µg/l	5.20 f	42.22 c
	50 µg/l	4.69 g	47.89 b
	100 µg/l	4.70 g	47.78 b
ZnO NPs	25 µg/l	8.10 bc	10.00 fg
	50 µg/l	7.30 de	18.89 de
	100 µg/l	6.90 e	23.33 d
Salicylic acid with Fe_3O_4 NPs	25 µg/l	6.90 e	23.33 d
	50 µg/l	4.30 g	52.22 b
	100 µg/l	3.80 h	57.78a
Control		9.00a	0.00h
L.S.D.	Nano	0.219	2.435
	concentration	0.205	2.281
	Nano × concentration	0.459	5.099

Table S3. Effect of different fungicide using poisoned media technique on the reduction percent of the linear growth of *S. cepivorum.*

Treatment	Concentration	Linear growth mean (cm)	Reduction percent (%)
Bellis® 38% WG	1 ppm	0.70 c	92.22 b
	10 ppm	0.50 d	94.44 a
	25 ppm	0.50 d	94.44 a
Topsin M, 70 %WP	50 ppm	0.90 b	90.00 c
	100 ppm	0.80 bc	90.37 bc
	200 ppm	0.50 d	94.44 a
BOGARD 250 g/l	1 ppm	0.70 c	92.22 b
	10 ppm	0.50 d	94.44 a
	25 ppm	0.50 d	94.44 a
Control		9.00 a	0.00 d
L.S.D.	Fungicide	0.061	0.675
	concentration	0.053	0.584
	Fungicide × concentration	0.105	1.168

Supplementary Figures

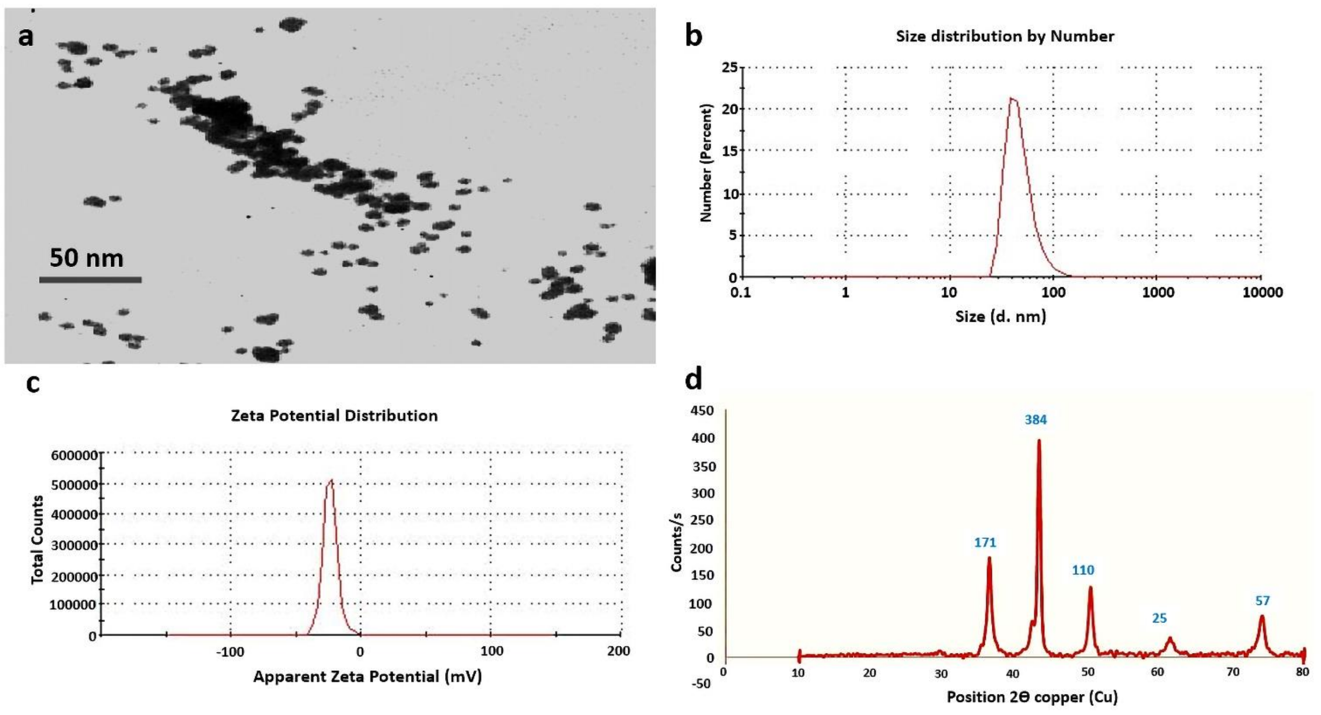

Figure S1. Characterization of Cu NPs: a) TEM image b) Particle size analysis c) Zeta potential analysis d) XRD pattern of Cu NPs

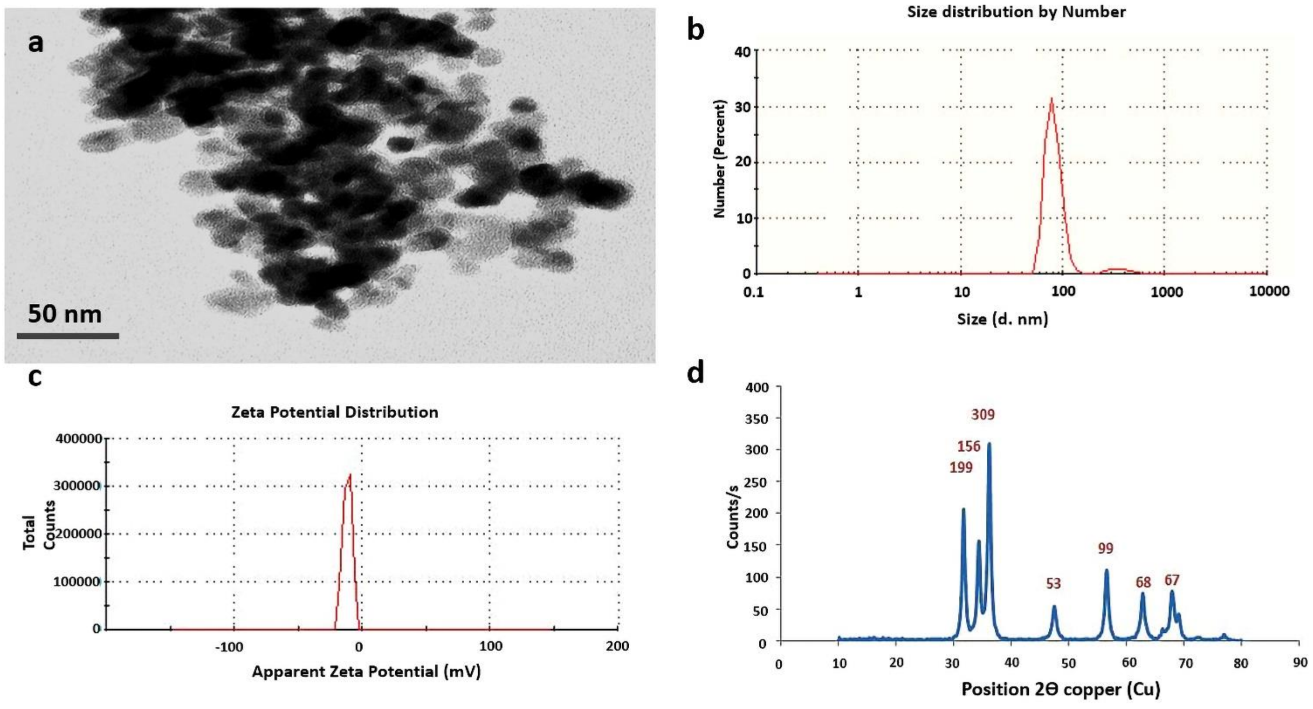

Figure S2. Characterization of Zn NPs: a) TEM image b) Particle size analysis c) Zeta potential analysis d) XRD pattern of Zn NPs

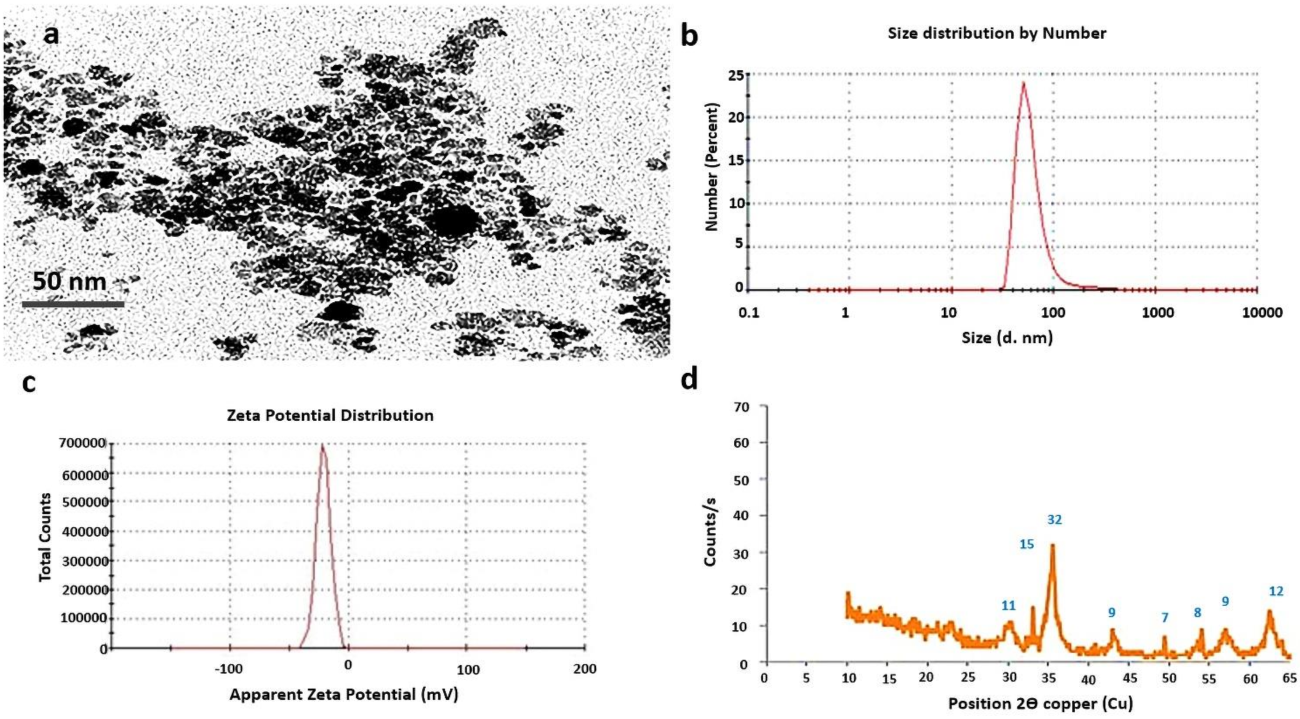

Figure S3. Characterization of Fe3O4 NPs: a) TEM image b) Particle size analysis c) Zeta potential analysis d) XRD pattern of Fe3O4 NPs

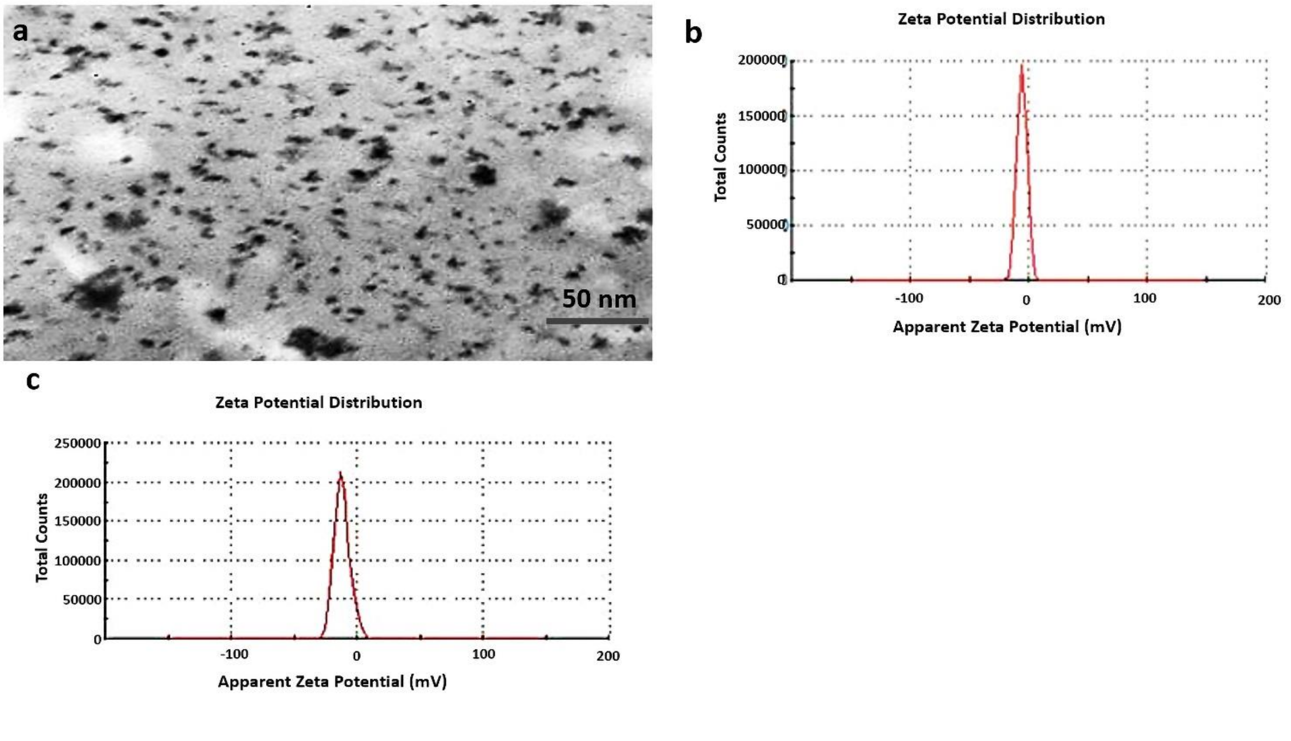

Figure S4. Characterization of SA- Fe NPs: a) TEM image b) Zeta potential analysis of SA c) Zeta potential of SA-Fe NPs

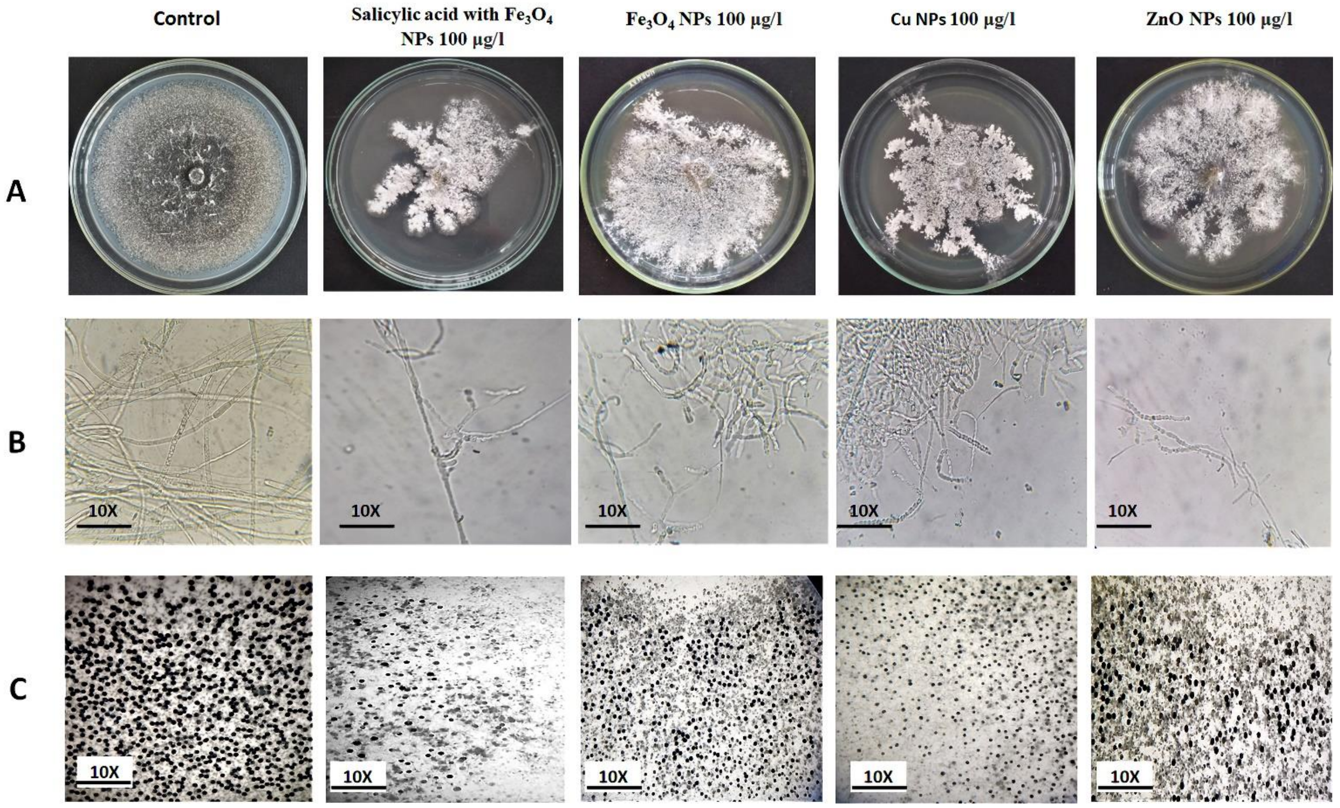

Figure S5. *In vitro* evaluation: A) the application of nanoparticles exerted control, inducing malformation in the morphology of mycelial growth, and preventing the formation of sclerotia. B) The impact of nanoparticles on mycelial malformation was observed under a light microscope. The mycelial malformation induced by nanoparticles was examined using stereomicroscopy.

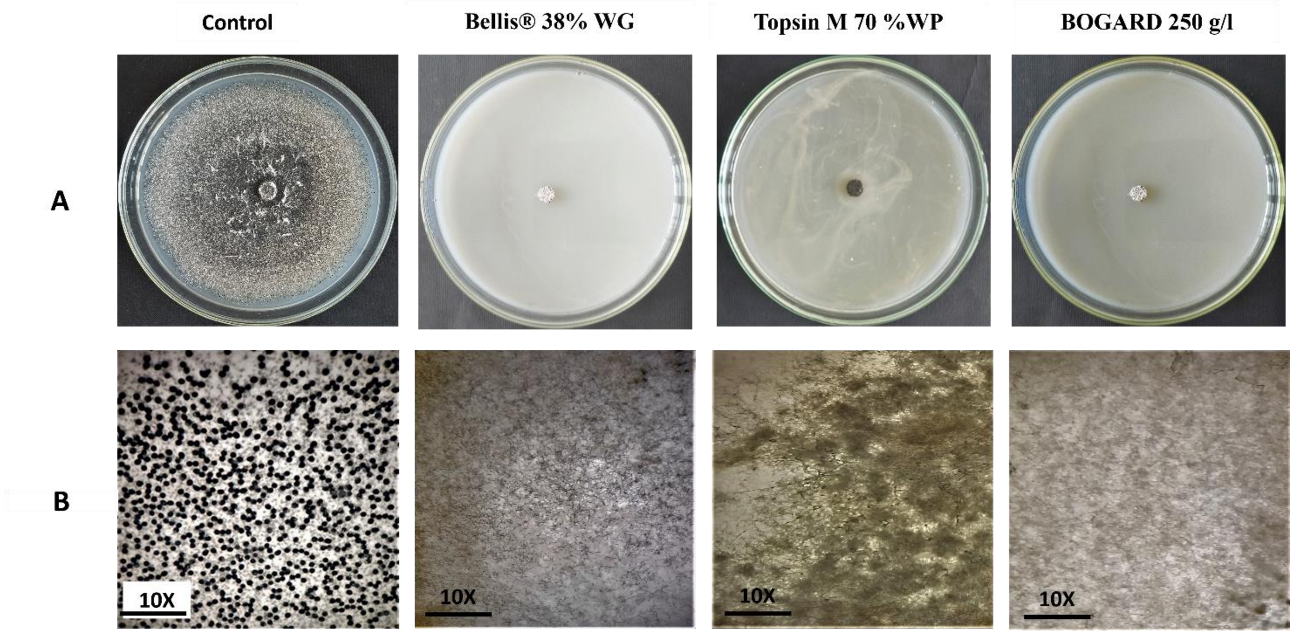

Figure S6. A) *In vitro* application of fungicides. Complete inhibition of mycelium and sclerotia was observed. B) Examination under a stereomicroscope revealed the effects of fungicide treatment on mycelial formation and sclerotia.
